# High, in Contrast to Low Levels of Acute Stress Induce Depressive-like Behavior by Involving Astrocytic, in Addition to Microglial P2X7 Receptors in the Rodent Hippocampus

**DOI:** 10.3390/ijms23031904

**Published:** 2022-02-08

**Authors:** Ya-Fei Zhao, Wen-Jing Ren, Ying Zhang, Jin-Rong He, Hai-Yan Yin, Yang Liao, Patrizia Rubini, Jan M. Deussing, Alexei Verkhratsky, Zeng-Qiang Yuan, Peter Illes, Yong Tang

**Affiliations:** 1 School of Acupuncture and Tuina, Chengdu University of Traditional Chinese Medicine, Chengdu 610075, China; zhaoyafeir@foxmail.com (Y.-F.Z.); pathlesswoods@126.com (W.-J.R.); zhangying802@126.com (Y.Z.); rong1215@126.com (J.-R.H.); yhy313@126.com (H.-Y.Y.); 2 The Brain Science Center, Beijing Institute of Basic Medical Sciences, Beijing 100850, China; yliao620@yahoo.com (Y.L.); zyuan620@yahoo.com (Z.-Q.Y.); 3 School of Medicine, University of South China, Hengyang 421000, China; 4 International Collaborative Centre on Big Science Plan for Purinergic Signalling, Chengdu University of Traditional Chinese Medicine, Chengdu 610075, China; patrizia.rubini@gmx.de (P.R.); Alexej.Verkhratsky@manchester.ac.uk (A.V.); 5 Molecular Neurogenetics, Max Planck Institute of Psychiatry, 80804 Munich, Germany; deussing@psych.mpg.de; 6 Faculty of Life Sciences, The University of Manchester, Manchester M13 9PL, UK; 7 Rudolf Boehm Institute for Pharmacology and Toxicology, University of Leipzig, 04109 Leipzig, Germany; 8 Key Laboratory of Sichuan Province for Acupuncture and Chronobiology, Chengdu University of Traditional Chinese Medicine, Chengdu 610075, China

**Keywords:** major depression, extracellular ATP, P2X7 receptor, hippocampus, rat, mouse

## Abstract

Extracellular adenosine 5′-triphosphate (ATP) in the brain is suggested to be an etiological factor of major depressive disorder (MDD). It has been assumed that stress-released ATP stimulates P2X7 receptors (Rs) at the microglia, thereby causing neuroinflammation; however, other central nervous system (CNS) cell types such as astrocytes also possess P2X7Rs. In order to elucidate the possible involvement of the MDD-relevant hippocampal astrocytes in the development of a depressive-like state, we used various behavioral tests (tail suspension test [TST], forced swim test [FST], restraint stress, inescapable foot shock, unpredictable chronic mild stress [UCMS]), as well as fluorescence immunohistochemistry, and patch-clamp electrophysiology in wild-type (WT) and genetically manipulated rodents. The TST and FST resulted in learned helplessness manifested as a prolongation of the immobility time, while inescapable foot shock caused lower sucrose consumption as a sign of anhedonia. We confirmed the participation of P2X7Rs in the development of the depressive-like behaviors in all forms of acute (TST, FST, foot shock) and chronic stress (UCMS) in the rodent models used. Further, pharmacological agonists and antagonists acted in a different manner in rats and mice due to their diverse potencies at the respective receptor orthologs. In hippocampal slices of mice and rats, only foot shock increased the current responses to locally applied dibenzoyl-ATP (Bz-ATP) in CA1 astrocytes; in contrast, TST and restraint depressed these responses. Following stressful stimuli, immunohistochemistry demonstrated an increased co-localization of P2X7Rs with a microglial marker, but no change in co-localization with an astroglial marker. Pharmacological damage to the microglia and astroglia has proven the significance of the microglia for mediating all types of depression-like behavioral reactions, while the astroglia participated only in reactions induced by strong stressors, such as foot shock. Because, in addition to acute stressors, their chronic counterparts induce a depressive-like state in rodents via P2X7R activation, we suggest that our data may have relevance for the etiology of MDD in humans.

## 1. Introduction

Major depressive disorder (MDD) arises from complex interactions between genetic, developmental, and environmental factors [1]. The lifetime prevalence estimates for MDD vary between 4.4% and 11%, with females having an approximately twofold higher disease risk than males [2]. Although the monoamine hypothesis of depression is still in the forefront of considerations, the role of P2X7 receptors (Rs) as a major etiological factor is gaining increasing acceptance.

The main function of P2X7Rs is to trigger IL-1β release and, thereby, to induce neuroinflammation [3,4,5,6]. Therefore, it was deduced that the activation of this receptor may cause MDD, which is reportedly accompanied by neuroimmunological alterations [7,8]. P2X7Rs respond to high local concentrations of adenosine 5′-triphosphate (ATP) that is supposed to be released into the extracellular space during inescapable stress and considered to be the main environmental factor instigating mood disorders in humans [9]. Based on this conviction, animal models of depression (e.g., tail suspension test [TST], forced swim test [FST], restraint stress, inescapable foot shock, and unpredictable chronic mild stress [UCMS]) utilize different stressors to induce a state termed “learned helplessness” [10,11,12].

The involvement of P2X7Rs in the development of depression-like behavioral changes has been suggested by threefold evidence: (1) a number of human epidemiological studies show that the single nucleotide polymorphism (SNP) *rs2230912* coding for Glu460Arg-P2X7R is associated with MDD [13] (for opposing data, see [14]); accordingly, humanized mice expressing this knocked-in polymorphic receptor showed alterations in their sleep quality resembling signs of a prodromal MDD state [15]. More recently, a better relationship between certain SNPs with depression severity were found when their interactions with childhood adversities and recent stressful life events were also taken into consideration [16]. (2) The genetic deletion of P2X7Rs in mice results in decreased behavioral despair and, consequently, reduced immobility in FST and TST, indicating an antidepressive phenotype [17,18]. (3) Especially, chronic stress models in rodents have convincingly born out the participation of P2X7Rs in depressive-like behaviors, in combination with immune changes (NLRP3 inflammasome assembly and activation, IL-1β release, microglia activation) and neuroplasticity impairments [19,20,21,22,23] (see also Section 3). In all of these studies, unpredictable, chronic mild stress or unpredictable, chronic stress was applied for the duration of a few weeks, and for the second half of each stress paradigm a P2X7R antagonist (Brilliant Blue G, A-438079, A-804598) was administered. The three antagonists unequivocally decreased the consequences of stressful stimulation. Brilliant Blue G had a similar effect in mice injected with lipopolysaccharide causing neuroinflammation, or reserpine causing inflammatory fibromyalgia and subsequently learned helplessness; both effects depended on the stimulation of the P2X7Rs by endogenous ATP [24,25]. The importance of these findings is underpinned by the assumed antidepressive properties of P2X7R antagonists in human MDD [26,27].

Although, originally, pathological neuronal functions were supposed to be the primary drivers of MDD, more recently, glial cells are expected to orchestrate this disease via glia-neuron interaction [5,28,29,30]. One of the reasons for this change in view is that the P2X7Rs were assumed to be located in the microglia and, to a lesser extent, in neuroglial cells, but not in neurons [4,31,32]. In addition, a significant contribution of astrocytes to MDD is accentuated by the finding that modification of astrocytic functions or a decreased number of astrocytes in the fronto-limbic and hippocampal regions is associated with depression [28,29,33]. It was reported that a defective release of ATP from astrocytes in the prefrontal cortex (PFC), which is one of the depression-relevant areas of the brain, prolonged the immobility times in FST; moreover, it also decreased the consumption of sucrose in the sucrose preference test signaling anhedonic behavior, a core symptom of depression [34,35]. In this respect, it is important to mention that the hippocampal-prefrontal pathway (a monosynaptic unidirectional projection) is highly sensitive to stress, which is a major precipitating factor for the symptoms of depression [36].

The aim of the present study was threefold. Firstly, we confirmed the participation of P2X7Rs in rodent models of MDD and searched for possible differences in the behavioral responses of rats and mice to inescapable stress. Secondly, in view of the emerging significance of the hippocampal CA1 area in this disease, we examined whether a range of MDD-relevant stressors modify the P2X7R sensitivity of CA1 astrocytes in different manners. Thirdly, we elucidated whether astrocytic P2X7Rs are involved in functional changes in all types of MDD-relevant stress models or only in some of them.

The results indicate that at lower levels of stress, the enhanced function of microglial P2X7Rs is a causative factor for depressive-like behavior, while at higher and longer lasting levels of stress (confirmed by protracted anhedonic reactions resulting in lower sucrose consumption) astrocytic P2X7Rs become involved in addition to their microglial counterparts.

## 2. Results

### 2.1. Involvement of P2X7Rs in Learned Helplessness

**Figure 1:**
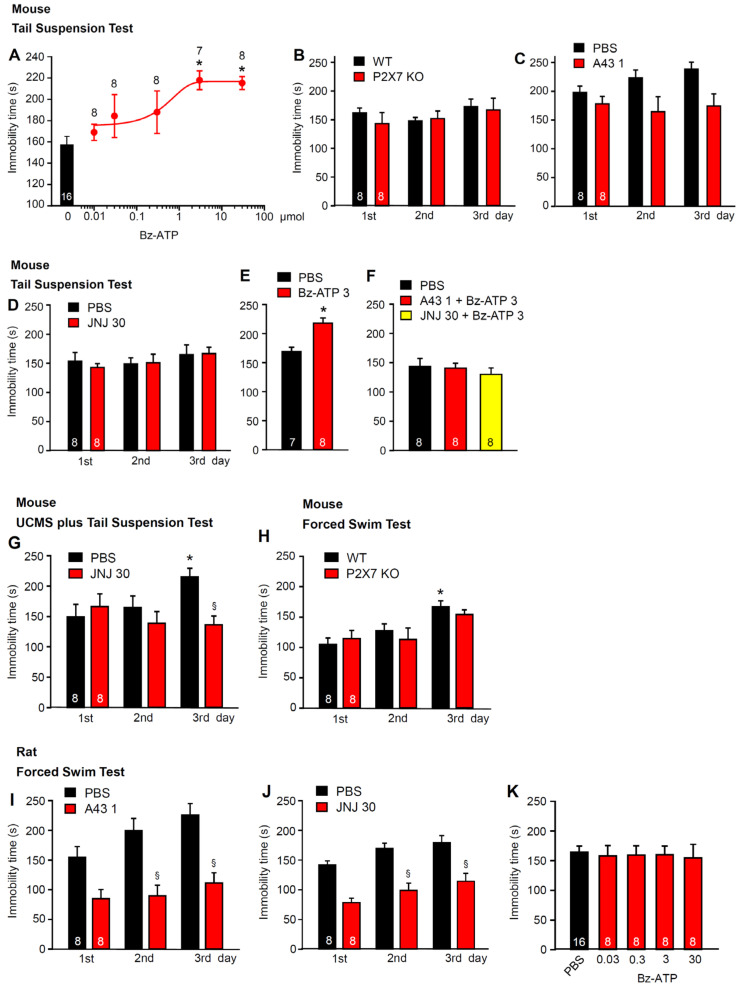
Effects of P2X7R agonists and antagonists in tests inducing learned helplessness in mice and rats. (**A**–**H**) Experiments in mice. (**A**) dose-dependent prolongation of the TST immobility time of mice by i.c.v. applied Bz-ATP, in comparison with i.c.v.-applied PBS. (**B**) no difference in the TST immobility time between the WT and P2X7R KO mice during repeated measurements on 3 successive days. No difference in the TST immobility time between mice injected i.c.v. with A-438079 (1 µM; **C**) or i.p. with JNJ-47965567 (30 mg/kg; **D**) in comparison with their PBS-injected counterparts. Prolongation of the TST immobility time by Bz-ATP (3 µM; i.c.v.; **E**) and its blockade by co-applied A-438079 (1 µM; i.c.v.) or JNJ-47965567 (30 mg/kg; i.p.; **F**). (**G**) the TST immobility time after CUMS was applied for 30 days. When, thereafter, the TST was measured for 3 successive days, on the third day there was a significant difference between PBS- and JNJ-47965567 (30 mg/kg; i.p.)-treated mice. (**H**) no difference in the FST immobility time between the WT and P2X7R KO mice during repeated measurements on 3 successive days. * *p* < 0.05; statistically significant difference from the PBS-injected controls (**A**,**E**) or the PBS-injected controls on the first day of application (**G**,**H**). (**E**, *t* = 4.238, *p* < 0.001; Student’s *t*-test). (**A**, F = 4.199, 30 µM, *p* = 0.004; 300 µM, *p* = 0.003; **G**, F = 3.827, 3rd day PBS, *p* = 0.039; one-way ANOVA followed by the Holm–Sidak test). (**B**, F_treatment_ = 0.261, F_genotype × treatment_ = 1.114, *p* = 0.571; **H**, F_treatment_ = 0.328, F_genotype × treatment_ = 0.568, *p* = 0.300; two-way ANOVA). ^§^ *p* < 0.05; statistically significant difference from the PBS-injected controls on the same day of application (**G**, F = 10.578, 3rd day, *p* = 0.008; one-way ANOVA, followed by the Holm–Sidak test). (**I**–**K**) experiments in rats. Decrease in the FST immobility time by A-438079 (1 µM; i.c.v.; **I**), or JNJ-47965567 (30 mg/kg; i.p.; **J**) in comparison with their PBS-injected counterparts. (**K**) Bz-ATP (0.03–30 µM; i.c.v.) fails to prolong the FST immobility time in the PBS-injected controls (one way ANOVA). ^§^ *p* < 0.05; statistically significant difference from the PBS-injected controls on the same day of application (**I**, F = 10.571, 2nd day, *p* = 0.001, 3rd day, *p* < 0.001; **J**, F = 14,975, 2nd day, *p* < 0.001, 3rd day, *p* < 0.001; one-way ANOVA followed by the Holm–Sidak test). The number of experiments is indicated throughout above each symbol (**A**) or within each column (**B**–**K**).

We first examined the involvement of P2X7Rs in the learned helplessness state induced by various stressful situations. It should be mentioned already at this stage that in the present series of in vivo experiments, the doses applied are expressed as “µmol”, whereas concentrations used in in vitro experiments are expressed as “µM” and mean, by convention, “µmol/L”. In behavioral experiments on mice, the immobility time in TST was prolonged, in a dose-dependent manner, 15 min after the i.c.v. injection of dibenzoyl-ATP (Bz-ATP; 0.03-300 µM; Figure 1A). The TST immobility time in untreated mice changed neither in the wild-type (WT) nor in the knockout (KO) animals (Figure 1B). No difference was observed either on the first day of testing, or on testing sessions carried out on the 2 consecutive days. In accordance with these results, none of the two P2X7R antagonists, A-438079 (1 µM; i.c.v.) (Figure 1C) or JNJ-47965567 (30 mg/kg; i.p.) (Figure 1D), had an effect. However, both antagonists prevented the Bz-ATP (3 µM; i.c.v.) effect when co-applied 15 min before behavioral testing (Figure 1E,F). Further, when TST was made after 4 weeks of CUMS, JNJ4795567 (30 mg/kg; i.p.) decreased the immobility time measured on the third day of measurement (Figure 1G). In contrast to the TST, the immobility time in the FST, as determined in acute experiments, increased from the first to the third day in the WT mice (Figure 1H). No such increase was observed in the KO animals. Nonetheless, the difference on the third day between the WT and KO mice was minimal and statistically not significant.

It was interesting to note that the P2X7R antagonists A-438079 (1 µM, i.c.v.) and JNJ-47965567 (30 mg/kg, i.p.), which did not practically interfere with the TST in mice, markedly decreased the immobility time of the FST in rats (Figure 1I,J). This agreed with the failure of Bz-ATP (0.03–30 µM) to exhibit any effect on the FST in rats (Figure 1K). In adult rats, usually the FST, rather than the TST is used as a standard stressor; the greater weight of rats over mice may damage the tails of the former animals during the suspension procedure. In our experiments, this was not a limiting factor because the young rats used by us had a comparatively low weight. Nonetheless, because the FST is the standard method stated in the literature for causing learned helplessness in rats, we applied this procedure in the experimental series to measure the agonist and antagonist effects (Figure 1I–K). We also suggest that in mice, the endogenous ATP concentration around the P2X7Rs in the hippocampus was non-saturating, while in the same area of rats, the ATP concentrations, because of their higher potency, saturated the P2X7Rs and, therefore, further activation was impossible (for the upper, steady-state phase of the “S”-shaped concentration-response curves, see Figure 3A–C and Section 3).

**Figure 2:**
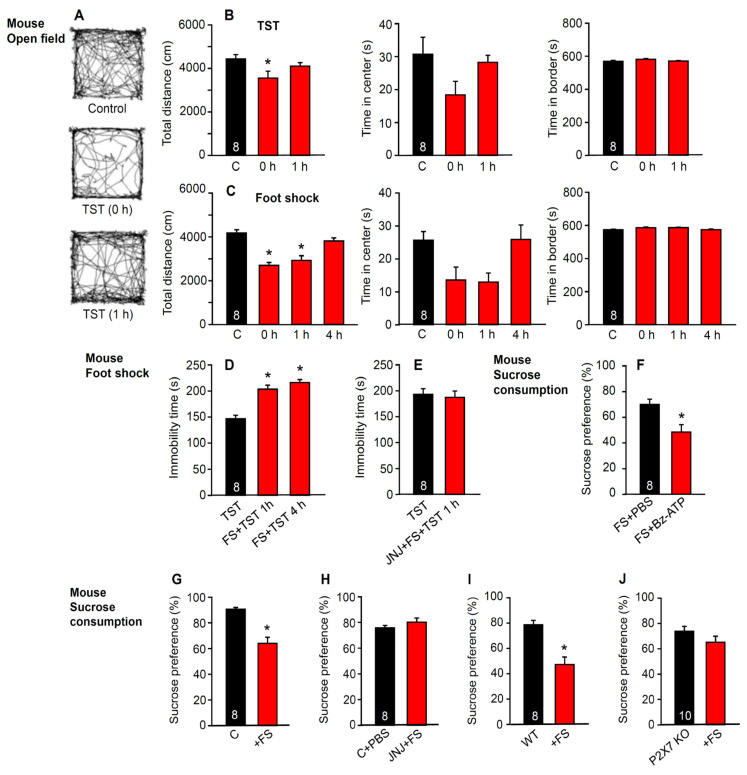
Effects of the TST and foot shock on the open-field behavior of mice; changes in the TST immobility time and sucrose consumption after foot shock and its antagonism by the blockade or genetic deletion of the P2X7Rs. (**A**) running traces of mice in the open-field apparatus under control conditions, immediately after TST, and 1 h after TST. The observation time was 10 min. (**B**) total running distance, time spent in the center, and time spent in the border of the open-field apparatus without TST (control), as well as immediately (0 h) and 1 h after TST. (**C**) total running distance, time spent in the center, and time spent in the border of the open-field apparatus without TST, as well as immediately (0 h), 1 h, and 4 h after foot shock. * *p* < 0.05; statistically significant difference from the three open-field parameters measured without the application of TST or foot shock (**B**, Total distance, F = 3.526, 0 h, *p* = 0.046; **C**, Total distance, F = 19.412, 0 h, *p* < 0.001, 1 h, *p* < 0.001) one-way ANOVA, followed by the Holm–Sidak test. (**D**) the TST immobility time measured without foot shock, or alternatively 1 or 4 h after foot shock. (**E**) the TST immobility time measured without foot shock or after the co-application of JNJ-47965567 (30 mg/kg; i.p.) and foot shock. * *p* < 0.05; statistically significant differences from the TST immobility time alone (**D**, FS + TST, F = 29.415, 1 h, *p* < 0.001, 4 h, *p* < 001; one-way ANOVA, followed by the Holm–Sidak test). Effect of i.c.v. Bz-ATP (10 µM) on the foot shock-induced modulation of sucrose preference when compared with the effect of the solvent PBS alone (**F**). Change in sucrose preference after foot shock (**G**) and the inhibition of this effect by JNJ-47965567 (30 mg/kg; i.p.) (**H**). In control experiments the solvent of JNJ-47965567 was applied before the foot shock (FS). The change in sucrose preference after foot shock (**I**) and no effect on the P2X7R KO animals (**J**). * *p* < 0.05; statistically significant difference from sucrose consumption after FS + PBS (**F**, FS + Bz-ATP, *t* = 3.102, *p* = 0.008) or untreated controls (**G**, *t* = 5.404, *p* < 0.001; **I**, *t* = 2.531, *p* = 0.024); Student’s *t*-test. The number of experiments is indicated in the first column of each set.

Next, we investigated the specificity of the TST and foot shock on the learned helplessness paradigm in mice (Figure 2A–C). This specificity was rather poor. Both the TST and foot shock decreased exploratory motility in the open-field apparatus and, in addition, although it was in a statistically non-significant but reversible manner, the time spent in the central areas was decreased. This suggests that the effects of the two stressors on exploratory behavior and fear perception, respectively, probably lasted for a shorter period (<4 h) than, e.g., the learned helplessness reaction (for comparison, see Figure 4F; <3 days). However, the immobility time in the TST, as measured 1 and 24 h after foot shock, was considerably prolonged, indicating an increase in learned helplessness by foot shock in comparison with the TST alone (independent groups of mice; Figure 2D). This change was due to P2X7R activation, as shown by the abolition of the facilitatory effect of foot shock by the selective P2X7R antagonist JNJ-47965567 (30 mg/kg, i.p.) (Figure 2E). The poor differentiation between depressive-like behavior and fear perception is not particularly unexpected because, in humans, MDD is usually accompanied by anxiety disorders and cognitive impairment [37,38].

In addition to measuring the TST after foot shock, we also investigated the anhedonic effect of this stressor on sucrose consumption and its dependence on P2X7R activation. In the first approach, it was shown that i.c.v. Bz-ATP (10 µM) further decreased the foot shock-induced change in sucrose preference when compared with the effect of phosphate buffered saline (PBS) alone (Figure 2F); this agrees with the observed prolongation of the TST immobility time by this agonist (Figure 1A). However, both the antagonism of the P2X7Rs by JNJ-47965567 (30 mg/kg i.p.; Figure 2G,H) and their genetic deletion (Figure 2I,J) prevented the decrease in sucrose preference induced by foot shock.

### 2.2. Immediate Modulation of Astrocytic Functions in the Hippocampus after Stimulation by Stressors

**Figure 3:**
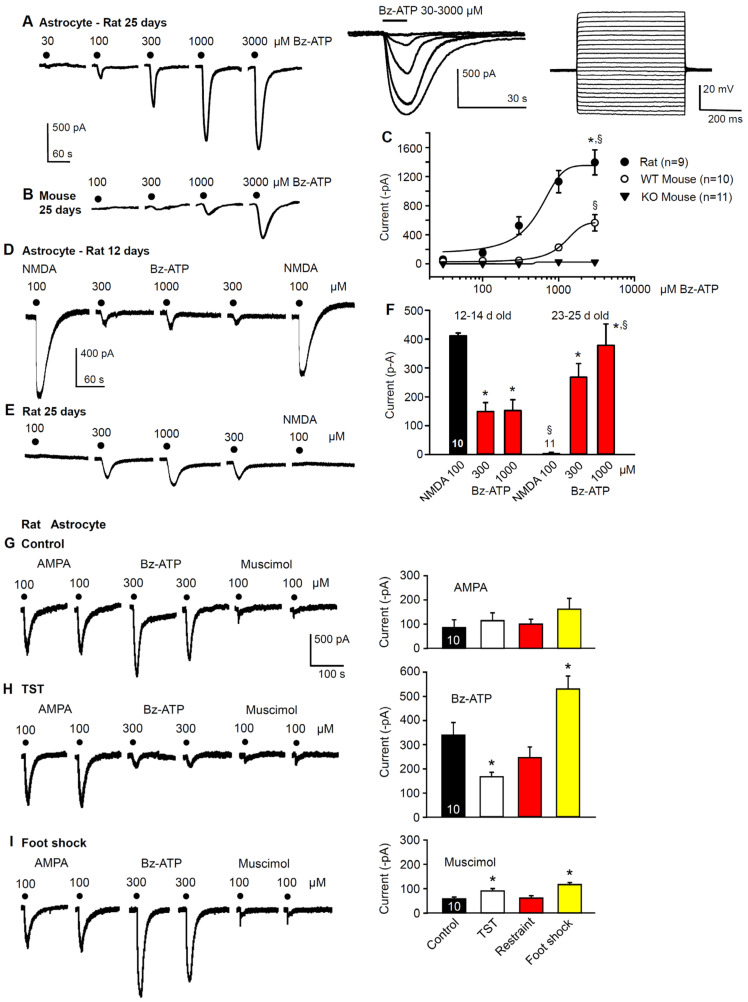
Bz-ATP-induced current responses in the hippocampal CA1 astrocytes of rats and mice, and their modification after the development of learned helplessness. Concentration-dependent increase of the current amplitudes (**A**) and current durations (**A**, **left inset**) caused by Bz-ATP, in preparations taken from 25-day old rats; representative tracings recorded at a holding potential of −80 mV. Bz-ATP (30–3000 µM) was applied every 3 min for 10 s. (**A**, **left inset**) superimposed current responses are shown at a longer time scale than those displayed in **A**. (**A**, **right inset**) injection of gradually increasing current pulses evoked only electrotonic changes in membrane potential and characterized astrocytes as belonging to the passive group. (**B**) concentration-dependent increase of the current amplitudes caused by Bz-ATP (100–3000 µM) in preparations taken from 25-day old mice. (**C**) plot of the Bz-ATP concentration against the current amplitude in astrocytes from rats, WT mice and P2X7R KO mice. Mean ± SEM of the indicated number of concentration-response relationships are shown. There was no response to Bz-ATP when hippocampal slices were prepared from the P2X7R KO mice. * *p* < 0.05; statistically significant difference from the effect of Bz-ATP (3000 µM) in WT mice (**C**, t = 4.033, *p* = 0.001; Student’s *t*-test). ^§^ *p* < 0.05; statistically significant difference from the effect of Bz-ATP (3000 µM) in the P2X7R KO mice (**C**, T = 144.00, *p* < 0.001; Mann–Whitney rank sum test). Current responses to NMDA (100 µM) and Bz-ATP (300, 1000 µM) of astrocytes prepared from 12-day (**D**) or 25-day old (**E**) rats; representative tracings. (**F**) mean ± SEM of the indicated number of experiments. * *p* < 0.05; statistically significant difference from the effect of NMDA (100 µM), in 12–14-day old astrocytes (**B**, F = 5.725, Bz-ATP 300 µM, *p* = 0.019, Bz-ATP 1000 µM, *p* = 0.014; one-way ANOVA, followed by the Holm–Sidak test), and from the effect of NMDA (100 µM) in 23–25-day old astrocytes (**B**, Bz-ATP 300 µM, *p* < 0.001, Bz-ATP 1000 µM, *p* = 0.002; one-way ANOVA, followed by the Holm–Sidak test, respectively). ^§^ *p* < 0.05; statistically significant difference from the effect of the corresponding NMDA (100 µM) and Bz-ATP (1000 µM) effects in the 12–14-day old group of astrocytes. (**B**, NMDA-NMDA, T = 165.00, *p* < 0.001, BzATP-BzATP, T = 165.00, *p* < 0.001; Mann–Whitney rank sum test). (**G**–**I**) current responses to AMPA (100 µM), Bz-ATP (300 µM) and muscimol (100 µM) in hippocampal CA1 astrocytes prepared from 20–25-day old rats. Representative tracings from brain slices taken from untreated (**G**, **left panel**), TST-treated (**H**, **left panel**) and foot shock-treated (**I**, **left panel**) rats. In the respective right panels, the mean ± SEM of identical experiments are shown for AMPA, Bz-ATP and muscimol. * *p* < 0.05; statistically significant difference from Bz-ATP (**H**, **right panel**, F = 12.285, TST, *p* = 0.029, foot shock, *p* = 0.018) and muscimol (**I**, **right panel**, F = 10.170, TST, *p* = 0.046, foot shock, *p* < 0.001) currents in control CA1 astrocytes (one-way ANOVA, followed by the Holm–Sidak test).

As mentioned in the introduction, the hippocampus, via its projections to the PFC, has been identified to be of critical importance in the development of MDD [36] and learned helplessness [39]. Here, we set out to identify the localization of these receptors at the astrocytes and neurons. For this purpose, hippocampal slices of rats and mice were used to patch-clamp astrocytes in the CA1 pyramidal cell region. In view of the known facilitation of P2X7R sensitivity towards the prototypic agonist Bz-ATP in a low X^2+^ medium (see Section 4), all of the measurements were carried out under these conditions. The neurons were discriminated from the astrocytes by comparing their respective voltage-current relationships; astrocytes, in contrast to neurons, failed to fire action potentials following the injection of a series of depolarizing current pulses into cells (Figure 3A, right panel of inset; see also Section 4). The 10 s superfusion with increasing concentrations of Bz-ATP (30–3000 µM) onto rat astrocytes caused current responses of gradually increasing amplitudes and prolonged duration (Figure 3A, and its left panel of inset). The 25 day-old rat astrocytes responded to progressively increasing Bz-ATP concentrations (30–3000 µM) with much larger current amplitudes at each concentration increment compared to mouse astrocytes of a similar age (Figure 3A,B). The concentration-response curves of the rat and WT mouse astrocytes confirmed the finding of different Bz-ATP-sensitivities and, in addition, showed that the P2X7 KO mice were completely insensitive to Bz-ATP in concentrations up to of 3000 µM (Figure 3C).

In the following experiments, we compared the current amplitudes induced by NMDA (100 µM) and Bz-ATP (300, 1000 µM), in very young (12–14) and young (23–25) day old rat astrocytes. Interestingly, both NMDA and Bz-ATP showed effects that depended on the age of the animals. Although the current response to NMDA was quite pronounced in the younger rats, it was completely absent in the older ones (Figure 3D–F). By contrast, Bz-ATP induced smaller currents in the younger than in the older rats. It should be mentioned already at this stage, that in experiments determining the P2X7R sensitivity before and after learned helplessness, a Bz-ATP concentration of 1000 µM was interposed between two 300 µM concentrations (Figure 3D). In these experiments, hippocampal slices of 20–25-day old rats were routinely used, to allow the TST, restraint and foot shock treatments in an age when the offspring had already stopped suckling from their mothers.

Next, the ionotropic glutamate receptor agonist AMPA (100 µM) was chosen instead of NMDA (100 µM), to reliably induce inward currents in 20–25-day old astrocytes; Bz-ATP (300 µM) activated the ionotropic P2 receptors (P2X7), and muscimol (100 µM) the ionotropic A-type γ-aminobutyric acid (GABA_A_) receptor (Figure 3G). Our aim was to find out whether there are some selective changes in the Bz-ATP-induced current without any major effect on currents through other ligand-gated ion channels. The TST, foot shock and restraint all modified the Bz-ATP currents, while the AMPA- and muscimol-induced currents did not change or increased only by a minor extent (Figure 3G–I). In fact, the effect of Bz-ATP was decreased by the TST and restraint but was increased by the foot shock.

**Figure 4:**
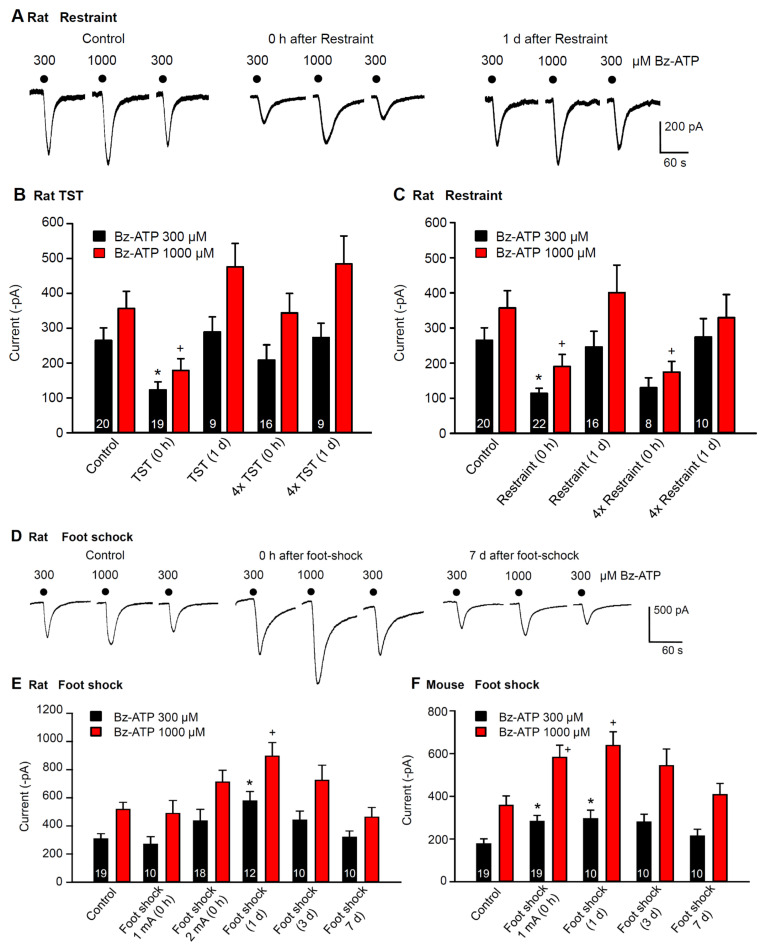
Effects of learned helplessness on Bz-ATP-induced current amplitudes in hippocampal CA1 astrocytes of rats and mice. (**A**) current responses of astrocytes to Bz-ATP (300, 1000 µM), prepared from rats which underwent no stress (control), or restraint stress, immediately or 1 day before preparing their hippocampal slices for recordings. Representative tracings. (**B**) mean ± SEM of current amplitudes in brain slices from rats unstressed (control) or stressed by TST. Current measurements were made immediately after TST (0 d), 1-day after TST (1 d), immediately after the last TST in a series of 4, applied on each consecutive day, and 1 day after a series of such stimulations. In this and all further experiments, two responses to Bz-ATP (300 µM) were averaged for further calculations. (**C**) mean ± SEM of current amplitudes in brain slices from rats unstressed or stressed by restraint. Measurements were immediately after restraint, 1-day after restraint, immediately after the last restraint in a series of 4, applied on each consecutive day, and 1 day after such a series of stimulations. (**D**) current responses of astrocytes to Bz-ATP (300, 1000 µM), prepared from rats which underwent no stress, or inescapable foot shock, immediately or 1, 3 and 7 days before preparing their hippocampal slices for recording. Representative tracings. (**E**) mean ± SEM of current amplitudes in brain slices from rats unstressed or stressed by foot shock. Current measurements were made immediately after foot shock with 1 mA or 2 mA current strength, as well as 1, 3 and 7 days after foot shock with 2 mA current strength. (**F**) mean ± SEM of current amplitudes in brain slices from mice unstressed or stressed by foot shock. Current measurements were made immediately after foot shock with 1 mA current strength, as well as 1, 3 and 7 days after foot shock. * *p* < 0.05; statistically significant differences from the effect of Bz-ATP (300 µM) in control preparations (**B**, F = 3.571, TST (0 h), *p* = 0.027; **C**, F = 4.852, restraint (0 h), *p* = 0.008; **E**, F = 4.473, foot shock (1 d), *p* = 0.001; **F**, F = 3.044, foot shock 1 mA (0 h), *p* = 0.025, foot shock (1 day), *p* = 0.033). ^+^ *p* < 0.05; statistically significant differences from the effect of Bz-ATP (1000 µM) in control preparations. (**B**, F = 5.073, TST (0 h), *p* = 0.005; **C**, F = 4.070, restraint (0 h), *p* = 0.033, 4× restraint (0 h), *p* = 0.030; **E**, F = 4.505, foot shock (1 d), *p* = 0.002; **F**, F = 4.318, foot shock (1 mA), *p* = 0.006, foot shock (1 d), *p* = 0.006). One-way ANOVA followed by the Holm–Sidak test in each case. The number of experiments is indicated throughout in each pair of columns (**B**,**C**,**E**,**F**).

By using the previously described protocol for the determination of P2X7R sensitivity, we compared the effects of the TST, restraint, and foot shock on rat hippocampal CA1 astrocytes (Figure 4A–E). Although the TST and restraint depressed the Bz-ATP (300, 1000 µM) currents immediately after inducing learned helplessness, inescapable foot shock facilitated these currents at the same time point. The effects of the TST and restraint disappeared 1 day after stimulation (Figure 4A–C); whereas, the effect of the foot shock persisted for a longer period (<7 days; Figure 4D,E). Next, we investigated whether TST or restraint, applied for 4 subsequent days, once per day, caused an equal or even larger inhibition than when applied only once on a single day; however, this was the case only with restraint stress (compare Figure 4B with Figure 4C).

In a similar experimental arrangement, the effect of foot shock also appeared to depend on the strength of the stimulating current applied; it reached statistical significance only when the current strength was increased from 1 to 2 mA (Figure 4E). Moreover, full recovery of the original sensitivity occurred after a significant delay. Foot shock, delivered to mice, caused similar facilitation of P2X7R sensitivity as in rats. In this case, a 1 mA current strength was sufficient to obtain the expected enlargement of Bz-ATP current amplitudes (Figure 4F).

### 2.3. No Immediate Change in Hippocampal Neuronal Functions after Stimulation by Stressors

**Figure 5:**
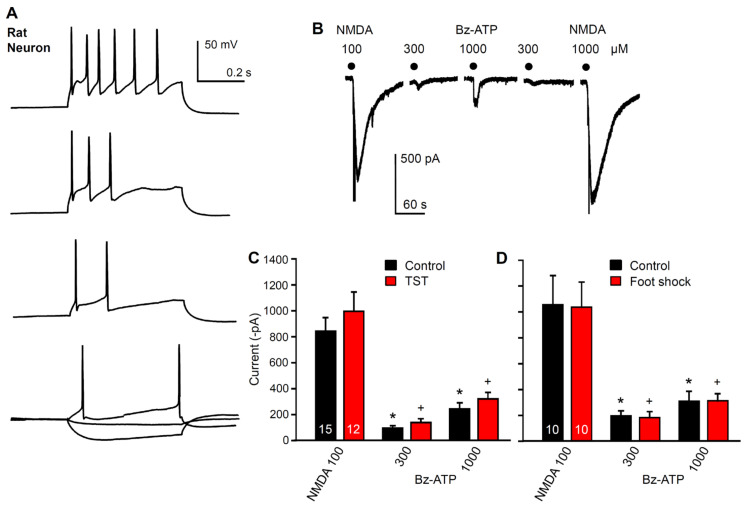
Effects of learned helplessness on NMDA and Bz-ATP-induced currents in hippocampal CA1 pyramidal neurons. (**A**) action potential firing caused by gradually increasing current injection into neurons discriminated thereby from astrocytes. Representative tracings. (**B**) the holding potential of the neurons was set to −70 mV and then NMDA (100 µM), and Bz-ATP (300, 1000 µM) was applied for 10 s every 3 min. Representative tracing. (**C**) there was no change in the NMDA- or Bz-ATP-induced current amplitudes after the TST, when compared with the unstressed (control) preparations. (**D**) there was no change in the NMDA- or Bz-ATP-induced current amplitudes after foot shock when compared with the unstressed preparations. * *p* < 0.05; statistically significant difference from the respective current response to NMDA under control conditions (**C**, F = 23.398, Bz-ATP 300, *p* < 0.001, Bz-ATP 1000, *p* < 0.001; **D**, F = 10.332, Bz-ATP 300, *p* < 0.001, Bz-ATP 1000, *p* = 0.002). ^+^ *p* < 0.05; statistically significant difference from the respective current response to NMDA after the TST or foot shock (**C**, F = 23.398, Bz-ATP 300, *p* < 0.001, Bz-ATP 1000, *p* < 0.001, **D**, F = 10.332, Bz-ATP 300, *p* < 0.001, Bz-ATP 1000, *p* = 0.002). There was no statistically significant difference between any of the agonist effects between that measured under control conditions or after the TST or foot shock, respectively. One-way ANOVA, followed by the Holm-Sidak test. The number of experiments is indicated in the first set of columns.

Another question to be answered was whether the electrophysiologically measured modifications of Bz-ATP current amplitudes in hippocampal astrocytes have any immediate effect on the function of neighboring neurons. To answer this question, we recorded the membrane currents from hippocampal CA1 neurons identified by their action potential firing in response to depolarizing current injection (Figure 5A). However, the Bz-ATP (300, 1000 µM)-induced currents did not change in amplitude immediately after the application of the TST (Figure 5B,C) or foot shock to mice (Figure 5B,D). NMDA (100 µM) produced large current responses, which also remained stable in spite of TST or foot shock stimulation.

### 2.4. No Change in the Density of P2X7 Receptor-Immunoreactivity in Hippocampal Astrocytes after Stimulation by Stressors

**Figure 6:**
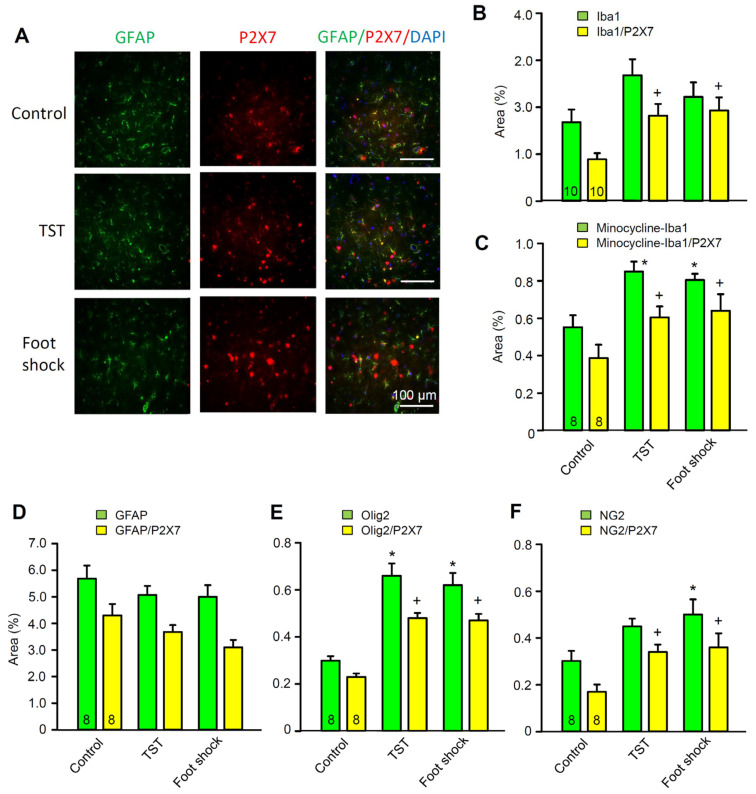
Effects of the TST and foot shock on P2X7R-immunoreactivities (IRs), with microglial (Iba1), astroglial (GFAP), and (pro)oligodendroglial (Olig2, NG2) markers as well as their co-localization with 4’,6-diamidino-2-phenylindol (DAPI) in the hippocampal CA1 region of 3-week old rats. (**A**) fluorescence microscopic pictures of immunopositive cells and DAPI-labelled nuclei. The astrocytic marker glial fibrillary acidic protein (GFAP; green fluorescence)-IR co-stains with the red fluorescent P2X7R-IR, and the blue fluorescent DAPI. Triple-labelling of all three IRs is displayed in the right row of the picture as shown. One representative snapshot obtained from the hippocampi of 8 animals. Scale bars, 100 µm. (**B**–**F**) The stained sections were examined under 400× magnification of the microscope over the whole image; the IF was evaluated with ImageJ software and, after subtracting the background, it was expressed as a % of the whole area. (**B**,**C**) the Iba1/P2X7-IR was increased by the TST and foot shock both with and without minocycline pretreatment of mice (see Section 4). However, minocycline largely decreased the number and density of the microglial cells. (**E**,**F**) oligodendrocytes and NG2 glial cells responded to the TST and foot shock in a similar manner as microglia did. (**D**) neither the GFAP- nor the GFAP/P2X7R-IR changed after TST or foot shock stimulation in comparison with the unstressed controls. * *p* < 0.05; statistically significant difference from the first column in a triad of columns in each panel (**B**, F = 2.635, *p* = 0.090; **C**, F = 9.681, TST, *p* = 0.002, foot shock, *p* = 0.005; **D**, F = 0.751, *p* = 0.484; **E**, F = 20.661, TST, *p* < 0.001, foot shock, *p* < 0.001; **F**, F = 4.258, TST, *p* = 0.093, foot shock, *p* = 0.032). ^+^ *p* < 0.05; statistically significant difference from the second column in a triad of columns in each panel (**B**, F = 6.206, TST, *p* = 0.016, foot shock, *p* = 0.010; **C**, F = 7.581, TST, *p* = 0.009, foot shock, *p* = 0.006; **D**, F = 3.265, *p* = 0.058; **E**, F = 41.398, TST, *p* < 0.001, foot shock, *p* < 0.001). One-way ANOVA, followed by the Holm–Sidak test in each case. The number of experiments is indicated in the first set of columns.

Eventually, we investigated the possible change of P2X7R density in rat CA1 microglial cells, astrocytes, and oligodendrocytes under resting conditions and after TST or foot shock by using immunohistochemistry (Figure 6A–F). However, it was quite clear that while the TST and foot shock increased the co-localization of P2X7R-immunofluorescence (IF) with the microglial marker Iba1 (Figure 6B), and the oligodendroglial markers Olig2 (Figure 6E) and NG2 (Figure 6F), they failed to modify the co-localization of P2X7R-IF with GFAP-IF (glial fibrillary acidic protein, astrocytic marker; Figure 6D). In addition, acute pretreatment with minocycline for 2 days largely depressed the basal co-expression of Iba1/P2X7, but did not inhibit the relative increases caused by the TST and foot shock (Figure 6C). This perfectly accorded with the finding that unpredictable chronic mild stress induced remarkable depressive and anxiety-like behaviors with simultaneous hippocampal microglial activation [40]. Although Olig2 is a general oligodendrocytic marker, NG2 glial cells (expressing the proteoglycan NG2) are oligodendrocyte precursor cells constituting a third type of neuroglial cell type [41,42]. In contrast to oligodendrocytes, they also occur in ample numbers in the grey matter such as the hippocampus. Hence, the immunohistochemistry suggests that both microglia and oligodendrocytes might equally contribute, via their P2X7Rs, to depressive-like behavior.

Further, we concentrated our attention on the astrocytes. The number of astrocytes in the field of view was 899.8 ± 146.0 under control conditions, 862.5 ± 131.6 after the TST, and 707.0 ± 90.5 after foot shock (*p* > 0.05; Kruskal–Wallis one-way ANOVA; see Section 4). Hence, the immunohistochemistry, performed 1 day after the TST or foot shock, did not show any changes in the number of P2X7R-IR astrocytes, or in the density of their P2X7R-staining. Therefore, it appears that the bidirectional regulation of the astrocytic P2X7R currents by learned helplessness caused by these stressors is due to a sensitivity change of the receptor, rather than to an up- or down regulation of its density. In contrast, P2X7Rs at microglia and NG2 cells increased in number and/or density after the TST and foot shock.

### 2.5. Metabolic Intoxication of Astrocytes in the Hippocampus Does Not Change the TST-Induced Depressive-Like Behavior

**Figure 7:**
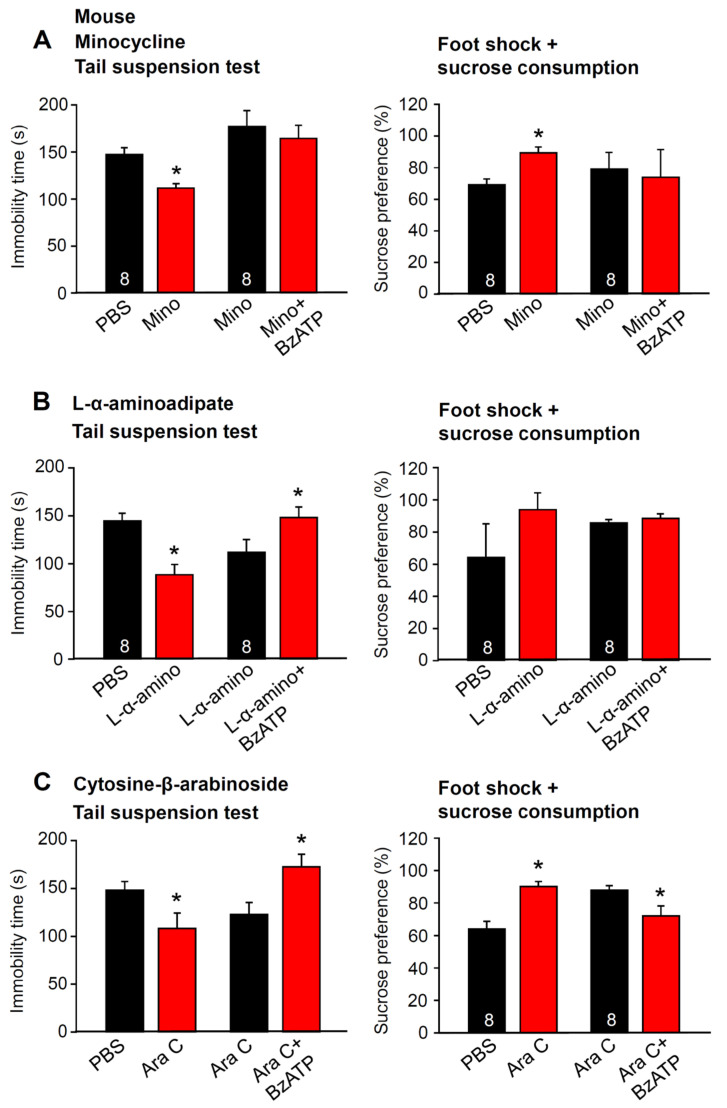
Effects of Bz-ATP in behavioral tests after the pretreatment of mice with drugs inhibiting microglial activation or interfering with astrocytic metabolism or oligodendrocytic proliferation. The immobility time in the tail suspension test and the sucrose preference after inescapable foot shock was measured in the left and right panels of **A**–**C**, respectively. Bz-ATP (10 µM) was applied unilaterally to the lateral ventricle of the brain. (**A**) blockade of microglial activation by minocycline (see Section 4) abolishes the prolongation of the TST immobility time (left panel) and the foot shock-induced decrease in sucrose preference (right panel), normally observed in response to Bz-ATP. (**B**) selective damage to astrocytes by L-α-aminoadipate does not change the prolongation of the TST immobility time (left panel) but abolishes the foot shock-induced decrease in sucrose preference (right panel), normally observed in response to Bz-ATP. (**C**) preferential blockade of oligodendrocytic proliferation by cytosine-β-arabinoside (Ara C), has no effect either on the prolongation of the TST immobility time (left panel), or the foot shock-induced decrease in sucrose preference (right panel), normally observed in response to Bz-ATP. * *p* < 0.05; statistically significant difference from the preceding column (**A**, **left panel**, Mino, t = 4.258, *p* < 0.001, BzATP, t = 1.065, *p* = 0.305; **A**, **right panel**, Mino, t = 3.913, *p* = 0.002, BzATP, t = 0.253, *p* = 0.804; **B**, **left panel**, α-Amino, t = 4.153, *p* < 0.001, BzATP, t = 2.109, *p* = 0.046; **B**, **right panel**, α-Amino, t = 1.265, *p* = 0.227, BzATP, t = 0.782, *p* = 0.447; **C**, **left panel**, Ara, t = 2.196, *p* = 0.045, BzATP, t = 2.707; *p* = 0.0170; **C**, **right panel**, Ara, t = 4.636, *p* < 0.001, BzATP, t = 2.366, *p* = 0.033); Student’s *t*-test. The number of experiments is indicated for each pair of columns.

The pretreatment of mice with minocycline, known to block microglial activation, decreased the immobility time in the TST and increased sucrose preference after foot shock (Figure 7A). Further, under these conditions the well-known promotion of the TST immobility time by i.c.v. Bz-ATP (10 µM; for choice of an appropriate dose see Figure 1A) was abolished (Figure 7A, left panel). Similarly, the Bz-ATP (10 µM)-induced sucrose preference decrease documented under control conditions (Figure 2F) was also abolished by using minocycline (Figure 7A, right panel). In partial contrast to these findings, the pretreatment of mice with the selective astrocytic toxin L-α-aminoadipate abolished only the effect of Bz-ATP on the foot shock-induced decrease in sucrose consumption (Figure 7B, right panel), but did not alter the Bz-ATP-induced prolongation of the immobility time in the TST (Figure 7B, left panel). Cytosine-β-D-arabinoside (AraC) pretreatment, which blocks with some preference oligodendrocyte proliferation, did not interact with the Bz-ATP (10 µM) effects either on the TST-prolonged immobility time (Figure 7B) or on foot shock-diminished sucrose consumption (Figure 7C). Hence, pharmacological damage of astrocytic functions in the hippocampus did not alter changes by the moderate and short acting stressor TST but interfered with those caused by the stronger and longer acting stressor of foot shock. In contrast to oligodendrocyte functions, microglial functions were involved in both types of depressive-like behavior.

## 3. Discussion

It was reported that the P2X7R KO mice exhibit an antidepressive phenotype in the TST, without changes in spontaneous locomotor activity [17,18]. However, Basso et al. [17] found a large difference in TST immobility between the KO and WT mice, while Csölle et al. [18] observed a less pronounced one. Furthermore, there was a marked difference between the FST-induced immobility of the KO and WT mice immediately after performing forced swimming, when determined by these two groups of authors, while at the same time point, Boucher et al. [43] found no disparity at all. However, on repeating the FST for 3 consecutive days, on the second and third day of testing, the duration of behavioral despair of the WT mice became more pronounced in comparison with that measured in P2X7R KO mice [43]. As mentioned above, in our experiments, neither the TST nor FST caused any difference during the 3 days of the testing period. This may be due to the complete knockout of all functional splice variants in our mouse model [44]. In previously generated KO mice used by the above authors, some immunologically and functionally active P2X7R splice variants evaded inactivation [45,46]. Recently, Metzger et al. [44] reported the generation of a conditional humanized *P2RX7* mouse. This *P2RX7* allele is accessible to spatially and temporally controlled Cre recombinase mediated inactivation; the KO mouse used in the present study was this *P2rX7^tm1.2Jde^* strain and its WT background.

Our experiments with P2X7R agonists and antagonists fully confirmed the data generated by the use of KO mice. In addition, they documented a Bz-ATP-induced potentiation of the TST immobility time in mice, although the FST-induced immobility time in rats was not affected. On the contrary, the selective P2X7R antagonists A-438079 and JNJ-47965567 shortened the TST immobility time in rats. Whereas, in acute models of depressive-like behavior, mice failed to respond to P2X7R antagonists, a chronic stressful procedure of CUMS rendered this species sensitive to JNJ-47965567. We concluded that the cause for this difference is the higher sensitivity of the rat P2X7R to agonists than that of its mouse orthologue. In fact, Bz-ATP activates human and rat recombinant P2X7Rs in the high micromolar and low millimolar range, whereas these mouse receptors are about an order of magnitude less sensitive to Bz-ATP [2,47]. Our electrophysiological experiments confirmed this difference in the potency of Bz-ATP at naïve astrocytic P2X7Rs of the two species (see Figure 3A–C). Thus, we suggest that a large amount of ATP released by a stressor may supramaximally activate local P2X7Rs in the rat hippocampus, while in the mouse hippocampus the same amount of ATP causes only submaximal activation sensitive to P2X7R antagonism.

In view of the unequivocal involvement of P2X7Rs in depressive-like states in rodents, it is no wonder that a lot of attention has been concentrated on microglia, the main bearer of this receptor type in the brain. Acute restraint stress has been reported to increase extracellular ATP, the inflammatory cytokine IL-1β, and the active form of the NLRP3 (NLR family pyrin domain containing 3) inflammasome in the hippocampus [19]. Similarly, UCMS leads to depressive-like behavior, and also resulted in higher protein levels of NLRP3 and IL-1β in the hippocampus of stress-exposed mice [48] and rats [40]. Microglia have been shown to be essential for these effects, because minocycline treatment, known to block the activation of microglia, inhibited the following engagement of NLRP3 inflammasome and the ensuing increased release of inflammatory mediators [40].

At this stage, we have put up the following question: Are P2X7Rs in astrocytes also responsible for learned helplessness? As already mentioned in the introduction, the modification of astrocytic functions in the fronto-limbic region was suggested to be associated with MDD. The observed changes included morphometric alterations of astrocytes in postmortem brain samples from individuals with depressive disorder or suicide completion [33,49]. In addition, animal studies have shown a causal relationship between the selective destruction of fronto-cortical astrocytes and depressive-like behavioral changes [5,50]. Neuronal projections extending from the CA1 region of the hippocampus to the PFC have been shown to be involved in emotional regulation [36]; persons suffering from MDD, or chronically stressed rats, displayed structural anomalies and aberrant functional coupling within the hippocampal-prefrontal circuits [36,39]. Based on these findings and the apparent tight relationship of P2X7R functions and MDD, we decided to search for possible changes in hippocampal CA1 astrocytic functions in animal models of MDD.

We assume that restraint and the TST represent relatively mild forms of stress, while foot shock is a more intensive and longer lasting stimulus. In fact, the TST immediately induced learned helplessness, while foot shock additionally caused enduring anhedonia as proved by a decrease in sucrose consumption. This may explain the findings that restraint and the TST decrease the amplitude of Bz-ATP-induced currents in CA1 hippocampal astrocytes, in contrast to foot shock, which facilitates these current amplitudes. Our experiments complement previous data showing a P2X2R-mediated antidepression in the PFC of mice subjected to social defeat stress [34]. The present experiments document a depression-like effect of hippocampal ATP via P2X7R activation in various behavioral models.

Several lines of evidence suggest that ATP gliotransmission is mainly carried out via exocytotic mechanisms. The release of ATP is depressed in transgenic mice, whose astrocytes express a dominant-negative (dn) form of the vesicular soluble N-ethylmaleimide-sensitive fusion protein attachment protein receptor (SNARE) [51,52]. In dnSNARE mice, the astrocytic ATP release was dysfunctional and a depressive-like behavior in the FST was evident [34]. In mice with selective genetic deletion of the vesicular nucleotide transporter (VNUT) in astrocytes, the fluoxetine-induced antidepressive behavior in the TST was impeded [53]. Alternative pathways of ATP release were also discussed, such as Calhm-2 channels [35] or connexin (especially Cx43) hemichannels and pannexin (Panx-1) channels. The expression of Cx43 immunoreactivity was reduced in the postmortem brains of patients suffering from MDD [54]. Conversely, the treatment of mice with antidepressants from diverse therapeutic classes increased Cx43 expression at both the mRNA and protein level [53]. In a good correlation with these findings, the application of Cx43-mimetic blocking peptides infused into the PFC of mice caused anhedonia in the sucrose preference test [53].

Astrocytes, to a large extent, define synaptic connectivity. Indirect effects are exerted by changes in astrocytic functions due to changes in brain homeostasis [55,56]. Astrocytes also directly modify synaptic transmission by releasing “gliotransmitters” (e.g., glutamate, GABA, and ATP) by exocytosis [51,52,57,58]. In addition, they may deliver ATP and ADP to the extracellular space by various non-exocytotic mechanisms [59,60]. It has been reported that astrocytic P2X7Rs release a number of signaling molecules (e.g., excitatory and inhibitory amino acids [4,31,61,62]) and, consequently, induce current responses at neighboring neurons; however, this may be responsible only for a part of the Bz-ATP current of CA1 pyramidal neurons. The residual responses are probably caused by direct stimulation of neuronal P2X7Rs [63] or to indirect effects caused by oligodendrocytic P2X7Rs [62]. Changes in the P2X7R-IR in oligodendrocytes was observed in this study but the mechanism of this effect was not investigated in the present paper.

In consequence, it was expected that the modified astrocytic responses to Bz-ATP immediately after applying a stressor would result in corresponding changes in the P2X7R sensitivity of the neighboring neurons by their signaling molecules. Nonetheless, this was not the case, indicating that P2X7R stimulation does not lead to short-term changes in neuronal function but causes the synaptic loss responsible for MDD with some delay, probably due to changes in brain homeostasis [64,65,66].

The postnatal development of laboratory rodents and humans occurs at a different pace; the rodent hippocampus proliferates maximally around P8, is still developing at P20, and reaches mature-like structures in the first to second postnatal month [67]. The same stage of maturation is achieved in humans only a number of years after birth. Therefore, our observations may have particular significance for early forms of MDD manifesting in childhood and also persisting later into adulthood [68].

Based on the electrophysiological and behavioral experiments, and on the immunohistochemistry results, it is concluded that relatively mild stress may cause MDD-like behavior in rodents by activating microglial P2X7Rs, while in the case of more pronounced stress, astrocytic P2X7Rs also become involved. This was directly confirmed—in agreement with the electrophysiology data—by the use of mice pretreated with the selective astrocytic toxin L-α-aminoadipate [69,70] or with minocycline [71], a selective inhibitor of microglial activation.

## 4. Materials and Methods

### 4.1. Animals

C57BL/6J male mice and male Sprague–Dawley rats (both from Chengdu Dossy Experimental Animal Co., Chengdu, China) were used for all of the experiments reported in this paper. In a few experiments, P2X7R KO mice (*P2rX7^tm1.2Jde^*; MGI:6203042) and their WT controls were used (generated by Jan M. Deussing, Max Planck Institute of Psychiatry, Munich, Germany). The animal study was reviewed and approved by the Institutional Review Board of the Chengdu University of Traditional Medicine, Chengdu, China (protocol code, DC1237, 1 January 2019).

### 4.2. Patch Clamp Recordings in Hippocampal Brain Slices

Rats and mice (10–15 or 20–25-day old, as indicated) were used for all of the experiments reported in this paper. Ample experience shows that patch-clamp recordings from neurons (but to a minor extent also from astrocytes) becomes increasingly difficult with an advanced age. To improve the comparability with the non-electrophysiological results, we chose, in most cases, 20–25-day old animals for patch-clamping. Because of the difficulties of patch-clamp approaches in older animals, we did not record membrane currents from astrocytes or neurons of the mice subjected to a chronic model of learned helplessness (UCMS).

The preparation of the brain slices and patch-clamp procedures were as described previously [4,61]. After decapitation, the brain was placed into ice-cold, oxygenated (95% O_2_ + 5% CO_2_) artificial cerebrospinal fluid (aCSF) of the following composition (in mM): NaCl 126, KCl 2.5, CaCl_2_ 2.4, MgCl_2_ 1.3. NaH_2_PO_4_ 1.2, NaHCO_3_ 25, and glucose 11. Hippocampal slices of the mice and rats were cut at a thickness of 200 μm (mice) or 300 µm (rats) by using a vibratome (VT1200S; Leica Biosystem, Muttenz, Switzerland).

To create low divalent cationic conditions (low X^2+^), MgCl_2_ was omitted from the medium and the CaCl_2_ concentration was decreased to 0.5 mM. The hippocampal slices were superfused in an organ bath with 95% O_2_ + 5% CO_2_-saturated low X^2+^ aCSF at 37 °C for 30 min, and then kept at room temperature (20–24 °C). Astrocytes in the CA1 region were visualized by using a 40× water immersion objective (LUMPlanFLN; Olympus, Japan). Patch pipettes were filled with an intracellular solution of the following composition (in mM): K-gluconic acid 140, NaCl 10, MgCl_2_ 1, HEPES 10, EGTA 11, Mg-ATP 1.5, Li-GTP 0.3; pH 7.3 adjusted with KOH. The pipettes were pulled by a horizontal micropipette puller (P-1000; Sutter Instruments, Novato, CA, USA) from the borosilicate capillaries.

Whole-cell current-clamp and voltage-clamp recordings were made using a patch-clamp amplifier (MultiClamp 700B; Molecular Devices, San Jose, CA, USA). CA1 astrocytes were discriminated from neurons by their failure to fire action potentials in response to a depolarizing current injection. In the current-clamp mode of recording, hyper- and depolarizing current pulses (−80 pA to 760 pA, in 60 pA increments) were injected into the cells. In some of the experiments, we used much larger hyperpolarizing pulses to inject current into the astrocytes (−610 pA to 760 pA, in 60 pA increments). Out of 40 non-spiking cells, 37 belonged to the passive class (typical astrocytes) and only 3 to the variably rectifying class; there were no in- or outwardly rectifying cells at all [72]. This is a clear distinction from microglia, which express, under resting conditions, an inwardly rectifying current pattern and acquire an additional outwardly rectifying current component only after their activation; for example, following facial nerve axotomy [73]. Oligodendrocytes similarly only express inwardly rectifying voltage-current relationships [74]. Furthermore, earlier experiments showed that when lucifer yellow (LY) diffused from the recording pipette into electrophysiologically-characterized astrocytes, these cells also stained immunohistochemically for the astrocytic marker S100β [72].

Then, in the voltage-clamp recording mode of the amplifier, the holding potential of astrocytes was set to −80 mV, and that of neurons to −70 mV. Agonists and antagonists were applied locally by means of a computer-controlled solenoid valve-driven pressurized superfusion system (VC^3^8; ALA Scientific Instruments, Farmingdale, NY, USA). The drug application tip touched the surface of the brain slice and was placed 100–150 µm from the patched cell. Agonists were applied for 10 s every 3 min, in a low X^2+^ solution. When the same concentration of an agonist was applied twice, the mean current response was calculated for statistical evaluation.

Astrocytic current amplitudes for Bz-ATP were measured in brain slices prepared from untreated animals, or from animals treated with a TST, foot shock (see below) or restraint stress (see Ref. [75]).

### 4.3. Behavioral Tests

#### 4.3.1. Tail Suspension Test (TST)

All of the behavioral experiments used 6-week old mice and 3-week old rats. They were suspended on a 55 cm high laboratory rack by adhesive tape positioned about 1 cm from the tail tip. The approximate distance between the animal’s nose and the operating floor was 20–25 cm. The animals were separated from each other by baffles to prevent mutual interference during the 6 min suspension time. The cumulative immobility time was recorded in each case. The TST was performed 10 min after injections of various drugs (see below) and only a single time in each animal. All of the behavioral tests were made at roughly the same time of the day (10:00 a.m. to 12:00 a.m.) and the evaluator was blind to the mice conditions.

#### 4.3.2. Forced Swim Test (FST)

The FST was performed in a clear glass cylinder filled with water (temperature, 23–25 °C). The volumes of the cylinders were different for rats (height, 65 cm; diameter, 30 cm; water level, 45 cm) and mice (height, 30 cm; diameter, 20 cm; water level, 15 cm). The mice or rats were gently placed in the tanks. The duration of immobility within the 6 min of observation was determined. The movement of the animals was video recorded and analyzed later. Following the swimming session, the mice and rats were removed from the water by their tails, gently dried with towels, and kept warm under a lamp in their home cages. They were considered to be immobile whenever they stopped swimming and remained floating passively, still keeping their heads above the surface of the water.

#### 4.3.3. Inescapable Foot Shock

The inescapable foot shock experiment was performed using a Learned Helplessness Test System (Shanghai Xin-Ruan Information Technology Co., Shanghai, China). The animals were placed in the apparatus and received electric foot shocks with a current intensity of 1 mA (mice) or 2 mA (rats) for 20 min; each stimulus lasted for 5–15 s with a random interval in-between. For electrophysiology 20–25-day old rats and mice were shocked before the preparation of their brains.

#### 4.3.4. Open Field Test

The apparatus consisted of a rectangular chamber (50 × 50 × 50 cm) made of white, high density, non-porous plastic. Before the open field test, mice received the TST or inescapable foot shock, as described above. Then, they were gently placed in the center of the chamber and their motility was recorded for 10 min. The total running distance, and the time spent in the center versus the periphery of the open field chamber were recorded by a camera connected to a computer using an automated video tracking program (EthoVision XT 9.0; Noldus, Wageningen, The Netherlands). The chamber was thoroughly cleaned with 95% ethanol, and dried prior to use and before subsequent tests, to remove any scent clues left by the previous subject.

#### 4.3.5. Sucrose Preference Test

The mice were singly caged for 3 days and given two 50 mL bottles of water and a 1% sucrose solution (*wt*/*vol*), respectively. The bottle positions were switched daily to avoid a side bias. Following a 22 h period of water and food deprivation, the preference for sucrose or water was determined overnight. Sucrose preference (%) was quantified as (vol sucrose/(vol sucrose + vol water)) × 100%.

#### 4.3.6. Unpredictable Chronic Mild Stress (UCMS)

Mice underwent 4 weeks of UCMS to establish a chronic model of depression. Two groups (prepared for PBS- or JNJ-47965567-treatment) of 8 animals each were subjected to different stimuli for different time durations on the successive days of the week as shown in Supplementary Table S1. These were, e.g., on the first week, restraint for 2 h (Tuesday); food and water deprivation for 5 h (Wednesday); cage tilt and no bedding for 5 h (Thursday); wet bedding for 5 h (Friday); altered light cycle for 5 h (Saturday); and altered light cycle and wet bedding for 5 h (Sunday) in the morning, partly combined with further manipulations in the afternoon. To ensure the unpredictability of the occurrence of the stimulation, these stressors were changed randomly per one or two stressors per day.

### 4.4. Stereotaxic Surgery

The mice or rats were anesthetized with isoflurane (5% induction; 2% maintenance; RWD Life Science, San Diego, CA, USA) and fixed on a stereotaxic platform (RWD Life Science). A stainless steel guide cannula (RWD Life Science) was implanted into the lateral cerebral ventricle (rats: −0.8 mm anterior-posterior and −1.5 mm lateral relative to bregma, 3.5 mm below the surface of the skull; mice: −1.0 mm anterior-posterior and −0.5 mm lateral relative to bregma, 2.2 mm below the surface of the skull) according to the stereotaxic coordinates. The cannula was secured to the skull using dental cement and small stabilizing screws. A dummy cannula (RWD Life Science) was inserted to maintain patency. At the end of the surgery, 5 mg/kg enrofloxacin (RWD Life Science) was administered subcutaneously (s.c.) to the animals to prevent postoperative infection, and all of the animals were placed on heating pads (37 °C) during surgery to keep their body temperature stable.

### 4.5. Drugs and Microinjections

The animals were allowed to recover from the surgical implantation of the cannulas for at least 7 days prior to the experiments. Bz-ATP and A-438079 (both from Sigma-Aldrich) were dissolved in PBS; Hyclone Labs., UT, Logan, USA). A-438079 (1 μM, 2 μL) and different concentrations of Bz-ATP (0.03–300 μM, 2 μL) dissolved in PBS were injected intracerebroventricularly (i.c.v.) to the lateral cerebral ventricle. JNJ-47965567 (30 mg/kg; Tocris Biosciences, Bristol, UK) was dissolved in 30% SBE-β-CD (MedChemExpress, Monmouth, NJ, USA) and was injected intraperitoneally (i.p.; Ref. [26]).

A 5 µL microsyringe was held by a microinjection system on the stereotaxic apparatus (all from RWD Life Science) and the injection was performed at a rate of 0.5 μL/min for mice and 1 μL/min for rats, respectively, through an injection tube via the guide cannula. The volume of each compound was 4 μL for rats and 2 μL for mice, respectively. The animals were fixed on a stereotaxic platform and anesthetized with 2% isoflurane during the microinjection; they recovered behaviorally 5–10 min after anesthesia. At the end of the infusion, the dummy cannula was retained at the injection site to allow sufficient diffusion.

A number of drug treatment protocols were used for the selective inactivation and ablation of the microglia, astrocytes or oligodendrocytes. Mice were injected i.p. with minocycline (40 mg/kg; Sigma-Aldrich, St. Louis, MO, USA) once daily over 2 days to inhibit the activation of the microglial cells [71]. On the third day, Bz-ATP was infused into the lateral ventricle (see above). To selectively abrogate the astrocytes, the astrocytic toxin L-α-aminoadipate (20 µg/mL stock, 5 µL at a constant rate of 0.5 µL/min; Sigma-Aldrich) was infused into the bilateral hippocampus [69]. The stereotactic coordinates for the positioning of the guide cannula into the bilateral hippocampus were -2 mm anterior-posterior and ±1.5 mm lateral to bregma, 1.5 mm below the surface of the skull. Then, 2 days were allowed to elapse and on the third day, Bz-ATP was injected through the same cannulas. The oligodendrocytes were abrogated by continuously infusing i.c.v. cytosine-β-D-arabinoside (Ara C; Sigma-Aldrich) at a dose of 120 µg/day via 14 day-osmotic minipumps (Alzet; Cupertino, CA, USA) with a flow rate of 0.25 µL/h [76]. On the fifteenth day, Bz-ATP was injected into the lateral ventricle. The control groups of mice obtained PBS, instead of minocycline, L-α-aminoadipate, or AraC via the same routs of injection and instead of Bz-ATP, PBS was applied into the lateral ventricle.

### 4.6. Fluorescence Immunohistochemistry

The untreated rats, and another drug-treated group of rats (3-weeks old in each case) 24 h after receiving the TST or foot shock were anesthetized with i.p.-injected 1% pentobarbital sodium (0.4 mL; Sigma-Aldrich). They were then perfused through the ascending aorta with saline followed by 4% paraformaldehyde (pH 7.2–7.4). Their brains were removed and post-fixed in the same fixative for 24 h and, afterwards, translocated into 30% of sucrose overnight. Then, the brain tissue was cut into 12 μm slices in a cryostat (Leica CM1860, Leica) and processed for immunostaining. Sections were blocked by adding 5% bovine serum albumin at 37 °C for 1 h prior to incubation with the primary antibodies. For double immunofluorescence staining, the sections were incubated with a mixture of antibodies for P2X7Rs (rabbit anti-P2X7 receptor polyclonal antibody, 1:200; Alomone Labs, Jerusalem, Israel), and GFAP (mouse anti-GFAP monoclonal antibody, 1:200; Proteintech, Chicago, IL, USA), Iba1 (mouse anti-Iba1 monoclonal antibody, 1:200; Abcam, Cambridge, UK), Olig2 (mouse anti-Olig2 monoclonal antibody, 1:400; Millipore, Burlington, MA, USA), or NG2 (mouse anti-NG2 monoclonal antibody, 1:400; Millipore) in PBS overnight at 4 °C. Then, incubation was continued for 2 h at 37 °C with a mixture of secondary antibodies (1:200 anti-mouse 488 [Proteintech] and 1:200 anti-rabbit Cy3 [Proteintech]), and counter-stained with DAPI (100 ng/mL; Sigma-Aldrich) for 5 min. The stained sections were examined with a Nikon Eclipse 50i (Nikon, Tokyo, Japan) fluorescence microscope, under 400× magnification. All of the images were taken with the same fluorescent settings, and subsequently adjusted equally for brightness and contrast to ensure accurate pathologic quantification. The yellow fluorescence density (co-expression level) of each image was analyzed using ImageJ software (version 1.52a). The immunofluorescence intensity was measured in whole image after subtracting the background, which is defined as the signal measured in an area devoid of specific immunostaining. The control experiments were performed without primary antibodies or by pre-absorption of the antibody with the immunizing peptides.

### 4.7. Data Analysis

All data were expressed as means ± SEM of *n* observations, where *n* means the number of cells taken from at least 3 animals, or in behavioral studies the number of animals, as appropriate. There was no a priori sample size calculation, but instead we have set the number of animals in behavioral and immunohistochemical studies to 8 (exception: Figure 6B with 10 animals), while the number of measurements in all of the other experiments was at least 10. SigmaPlot 14.0 was used for the statistical evaluation and construction of the concentration and dose-response curves (3 parametric Hill plot). We tested for and found that, when using parametric tests, all sampled distributions satisfied the normality and equal variance criteria. Multiple comparisons between data were performed in case of their normal distribution by one-way ANOVA followed by the Holm–Sidak test. Multiple comparisons between the data were performed in case of their non-normal distribution, using the Kruskal–Wallis ANOVA on ranks, followed by the Tukey’s test. A two-way ANOVA followed by the Dunn’s test was performed to compare data obtained in the KO and WT mice in Figure 1B,H. Post-hoc tests were run only if F or H achieved *p* < 0.05. Two data sets were compared using the parametric Student’s *t*-test or the non-parametric Mann–Whitney rank sum test, as appropriate. A probability level of 0.05 or less was considered to be statistically significant.

## Data Availability

All data can be sent on request from the corresponding author.

## Supplementary Materials

The following supporting information can be downloaded at: https://www.mdpi.com/article/10.3390/ijms23031904/s1.
