# Supplementary material for: High, in Contrast to Low Levels of Acute Stress Induce Depressive-like Behavior by Involving Astrocytic, in Addition to Microglial P2X7 Receptors in the Rodent Hippocampus
Source: Int J Mol Sci. 2022 Feb 8;23(3):1904. doi: 10.3390/ijms23031904 (PMC8836505; doi:10.3390/ijms23031904)
Supplement: Supplementary file 1 [file ijms-23-01904-s001.zip › ijms-1506994-supplementary.pdf]

Supplementary Table S1 Manipulations during a 4 week exposure of mice to unpredictable chronic mild stress (CUMS).

	time	Monday	Tuesday	Wednesday	Thursday	Friday	Saturday	Sunday
week1	**8 a.m.**						altered light cycle	altered light cycle+wet bedding
	**9 a.m.**			food and water deprivation	cage tilt+ no bedding	wet bedding		
	**10 a.m.**							
	**11 a.m.**							
	**12 a.m.**		restraint (0.5 h)					
	**1 p.m.**							
	**2 p.m.**				forced bath			
	**3 p.m.**					no bedding overnight	social stress	no bedding overnight
	**4 p.m.**							
week2	**8 a.m.**							
	**9 a.m.**			cage tilt+wet bedding		altered light cycle		
	**10 a.m.**						restraint (0.5h)	
	**11 a.m.**							
	**12 a.m.**	cage tilt						
	**1 p.m.**							
	**2 p.m.**				cage tilt+food and water deprivation	social stress+wet bedding overnight		
	**3 p.m.**			foot shock+wet bedding				no bedding overnight
	**4 p.m.**	forced bath						
week3	**8 a.m.**							
	**9 a.m.**				wet bedding			cage tilt+altered light cycle
	**10 a.m.**			altered light cycle				
	**11 a.m.**	forced bath	cage tilt+ food and water deprivation					
	**12 a.m.**					forced bath		
	**1 p.m.**							
	**2 p.m.**				foot shock+noise(120 Hz)	no bedding		
	**3 p.m.**							noise (120 Hz)
	**4 p.m.**							
week4	**9 a.m.**				cage tilt+wet bedding	forced bath	altered light cycle	
	**10 a.m.**							
	**11 a.m.**	wet bedding						
	**12 a.m.**		restraint (0.5 h)					
	**1 p.m.**							
	**2 p.m.**					no bedding	noise(120Hz)	
	**3 p.m.**		no bedding overnight					
	**4 p.m.**				food and water deprivation			
